# A parosmia severity index based on word-classification predicts olfactory abilities and impairment

**DOI:** 10.1007/s00405-023-07893-2

**Published:** 2023-03-11

**Authors:** Thomas Hörberg, Rumi Sekine, Clara Overbeck, Thomas Hummel, Jonas K. Olofsson

**Affiliations:** 1 Department of Psychology, Stockholm University, Albanovägen 12, 114 19 Stockholm, Sweden; 2 Department of Otorhinolaryngology, Smell and Taste Clinic, TU Dresden, Germany; 3 Department of Otorhinolaryngology, Jikei School of Medicine, Tokyo, Japan

**Keywords:** Olfactory disorder, Olfactory semantics, Natural-language processing, Behavioral categorization, Parosmia severity index

## Abstract

Parosmia is an olfactory disorder that involves distortions of specific odors that may co-occur with anosmia, loss of smell of other odors. Little is known about which odors frequently trigger parosmia, and measures of parosmia severity are lacking. Here, we present an approach to understand and diagnose parosmia that is based on semantic properties (e.g., valence) of words describing odor sources (“fish”, “coffee”, etc.). Using a data-driven method based on natural language data, we identified 38 odor descriptors. Descriptors were evenly dispersed across an olfactory-semantic space, which was based on key odor dimensions. Parosmia patients (*n* = 48) classified the corresponding odors in terms of whether they trigger parosmic or anosmic sensations. We investigated whether these classifications are related to semantic properties of the descriptors. Parosmic sensations were most often reported for words describing unpleasant odors of inedibles that are highly associated to olfaction (e.g., “excrement”). Based on PCA modeling, we derived the Parosmia Severity Index*—*a measure of parosmia severity that can be determined solely from our non-olfactory behavioral task. This index predicts olfactory-perceptual abilities, self-reported olfactory impairment, and depression. We thus provide a novel approach for investigating parosmia and establishing its severity that does not require odor exposure. Our work may enhance our understanding of how parosmia changes over time and how it is expressed differently across individuals.

**Supplementary Information:**

The online version contains supplementary material available at 10.1007/s00405-023-07893-2.

## Introduction

Parosmia is an olfactory disorder (OD) where odor and flavor perception is distorted; a variety of odor stimuli may trigger unpleasant sensations (e.g., smelling freshly brewed coffee, but experiencing a rotten smell, see, e.g., [26]). The condition can have extensive negative impacts on many aspects of everyday life [3]. It has been suggested to be triggered by a specific set of odor-active molecules, indicating that the distortion at least in part occurs on the receptor level [25]. Parosmia is, together with phantosmia (experiencing an odor without the presence of a triggering source), referred to as qualitative OD. Qualitative OD are less well understood than quantitative OD, such as hyposmia—reduced olfactory ability—and anosmia—complete loss of smell. Parosmia often occurs simultaneously with smell loss, as the distorted sensations may appear in the recovery phase from an olfactory deficit, especially when it is caused by a viral infection in the upper respiratory system [11]. Olfactory disorders are common in the general population, especially in the older age-groups, but are often not diagnosed. The prevalence of smell distortions in the adult population may range from 1 to 5% [16, 23]. The recent increase of patients with COVID-19 suffering from parosmia has led researchers to focus on which molecules may be responsible for the most common parosmia triggers [24–26]. For example, the involvement of sulfur-based molecules may explain why coffee, toast, and fried meat are among the most commonly reported parosmia triggers in COVID-19 [26]. If common parosmia triggers tend to share molecular structure, they might very well share semantic features as well (e.g., different types of vegetables, such as those high in starch, share molecular structure [4]). Knowledge about the relationships between semantic features and parosmic sensations can advance our understanding of the disorder, and may be used to develop more fine-grained measures of parosmia severity.

In the present study, we introduce a new approach toward these two goals that is based on powerful quantitative methods developed in the field of linguistics. We investigate whether the prevalence of parosmic sensations of particular odors can be explained by the semantic properties (e.g., valence) of the terms describing those odors, and then develop a measure of parosmia severity on the basis of our findings. We have in the last few years learnt more about olfactory vocabularies; odor-associated words and their associations [12, 13, 20]. Here, we ask parosmia patients to classify a set of source-based odor descriptors (i.e., words describing odors by reference to their source, such as “fish”, “coffee”, etc.) in terms of whether they elicit parosmic sensations or not, and whether they fail to evoke any olfactory sensation. The descriptors were selected with the aim of being representative of all types of odors that people talk about in everyday situations, and were thus selected to be evenly dispersed across an olfactory-semantic space. Descriptors were identified using a new data-driven method, proposed by Hörberg et al. [12], and based on natural written texts. This method identifies odor-associated descriptors based on how strongly they are associated with olfaction. Olfactory association is estimated based on a large collection of written texts from the web, a so-called text *corpus*. In this corpus, the distribution of words across olfactory and non-olfactory contexts is calculated (see [13]). Then, identified olfactory descriptors are used to derive a quantitative “olfactory-semantic space” which includes the properties and relationships of olfactory-related descriptors used in natural written language. This analysis is conducted with a language model that represents semantic properties of descriptors as vectors called *word embeddings*. The model represents semantic similarities between words as distances in the multi-dimensional space of the embeddings (see, e.g., [6] for an introduction to word embeddings). To capture olfactory meanings of our descriptors, the model is specifically trained on sentences related to olfaction and gustation. We then select a subset of descriptors that are evenly dispersed across this olfactory-semantic space and use these descriptors to ask parosmia patients about perceptual distortions of the corresponding odors. To investigate whether any of the semantic dimensions of our olfactory-semantic space are related to perceptual distortions, we evaluate these perceptual distortion ratings against a number of semantic variables of the selected descriptors. If parosmic experiences are related to any of the semantic properties represented in the olfactory space, some of these semantic variables should predict the frequency with which a corresponding odor is perceptually distorted.

On the basis of principal component analysis (PCA) of our data, we finally derive the *parosmia severity index—*a measure of the severity of parosmia. We evaluate this measure against a set of other, independent participant variables (such as olfactory threshold sensitivity and subjective impairment severity) that are diagnostic of parosmia severity. If our parosmia severity index indeed is diagnostic of parosmia severity, it should be related to some or all of these participant variables.

## Materials and methods

### Participants

The prospective study was performed at the Smell & Taste Clinic of the Department of Otorhinolaryngology at the TU Dresden. It was approved by the local ethic committee of TU Dresden (application number BO-EK-7802020). A total of 48 patients were included in this study (30 women, 18 men; mean age 45 years, SD 12; range 21–70 years). All patients presented themselves as having OD in combination with parosmia. All participants received a standardized structured medical history [30] which included age, sex, body mass index (BMI), cause, and duration of OD. All participant received nasal endoscopy before sensory testing.

### Procedure

#### Questionnaire on qualitative olfactory dysfunction

With regard to parosmic olfactory distortions, patients received a questionnaire that included questions about its duration (in months), intensity (on a visual analogue scale ranging from 0 [extremely weak, not present] to 10 [very intense]), pleasantness (on a visual analogue scale ranging from − 5 [very unpleasant] to + 5 [very pleasant]), frequency (daily, not daily), and weight change or other significant consequences (present, not present). A score estimating the degree of parosmia was calculated as the weighted sum of parosmia intensity, parosmia frequency, and significant parosmia consequences. Patients also rated the severity of the experienced impairment of their dysfunction (on a visual analogue scale ranging from 0 [no severity] to 10 [maximum severity]). Patients were asked about specific triggers of the parosmia, and they described the quality of the parosmic distortions. In addition, they filled in a questionnaire asking, for 38 named odors, whether they have no smell, smell like they always do, or whether they were qualitatively distorted. They could also indicate that they were unfamiliar with a particular odor.

#### Sensory testing

Olfactory function of all participants was tested using the reliable and validated Sniffin’ Sticks test battery (Burghart, Wedel, Germany). They were tested for phenyl ethyl alcohol (PEA) threshold (T), odor discrimination (D), and odor identification (I) as described previously [10, 22]. The aggregate TDI score serves as a measure of general olfactory-perceptual ability and was used to classify participants as anosmic, Hyposmic, or normosmic.

#### Depression scale, ADS-L scale

The ADS-L scale is a short self-report scale designed to measure depressive symptomatology in the general population [21]. The scale consists of 20 questions based on symptoms associated with depression. Participants were asked to answer based on how frequently they felt symptoms during the past week. The score ranged from 0 to 60.

#### Importance of olfaction questionnaire

The questionnaire on individual significance of olfaction refers to the role of one’s sense of smell in daily life [5]. Participants were asked to answer 20 questions with three subscales: association with olfactory sensations, application of the sense of smell, and the readiness to draw consequences from the olfactory perception.

#### Semantic variables

To investigate which semantic dimensions are associated with parosmia impairment, we evaluated our behavioral data against the descriptors' word valence and word arousal associations, as well as their associations to the olfactory and gustatory modalities. We were primarily interested in whether variability in the behavioral responses of the parosmia patients can be explained by any of these semantic properties. In particular, as previous research has shown that odors, both perceptually and semantically, are primarily differentiated in terms of valence, on the one hand, and edibility, on the other [12, 14, 15, 31], we wanted to see if these dimensions play a role in parosmia reports.

Word valence and arousal ratings were obtained from the data set published by Warriner et al. [29]. It contains ratings for more than 14,000 words, rated on 1–9 grade Likert scale. High valence ratings correspond to feelings of being *happy, pleased, satisfied, contented,* or *hopeful*, and low ratings to feelings of being *unhappy, annoyed, unsatisfied, melancholic, despaired, or bored*. High arousal ratings correspond to feelings of being *stimulated*, *excited*, *frenzied*, *jittery*, *wide-awake*, or *aroused*, and low ratings to feelings of being *relaxed*, *calm*, *sluggish*, *dull*, *sleepy*, or *unaroused* [29]. We extracted valence and arousal ratings for those words that overlapped with our final selection of 38 descriptors (see below). In total, 163 (82%) of our descriptors were assigned a valence and an arousal rating.

Olfactory and gustatory modality associations come from the ratings collected by Lynott et al. [19]. This data set contains ratings of almost 40,000 words. These words were rated on a 1–5 Likert scale with respect to the extent to which the objects that the words designate are experienced by smelling and by tasting [19]. These ratings thus estimate how strongly the words are associated with gustatory and olfactory modalities. Gustatory modality ratings serve as a proxy for edibility, because the associations to taste are presumably caused by experiences related to eating. We extracted ratings for words that overlapped with our identified descriptors, resulting in the assignment of ratings to 178 (89%) of our descriptors.

#### Descriptor selection

The olfactory descriptors evaluated in this study were identified on the basis of a method proposed by Hörberg et al. [12]. This method automatically identifies odor descriptors on the basis of their distribution in a collection of natural texts from the web (see Supplementary Materials Sect. 1 for a description of this text corpus). Descriptors can differ with respect to their olfactory association and specificity: whereas some descriptors first and foremost are used to express odor qualities (e.g., *smelly*), and therefore are strongly associated with olfaction, others are used in a broader range of contexts (e.g., *sharp* can be used to describe cheese smell, but also corners, knives and sounds), and thus only weakly associated with olfaction. Descriptors can also either be used for a narrow range of odors or flavors (e.g., *flowery*) and thus be specific, or they can apply to a wider range of odor qualities (e.g., *strong*) and thus be unspecific. Here, we use the corpus-based measures of olfactory association and olfactory specificity proposed by Iatropolous et al. [13]—the *Olfactory Association Index* (OAI) and the *Olfactory Specificity Index* (OSI) to identify 200 olfactory descriptors. The semantic distances between the identified descriptors were then calculated on the basis of word embeddings from a language model that was trained on olfactory contexts from the web. As mentioned above, this model represents semantic distances as distances in the multi-dimensional space of the embeddings. We then used the resulting distances to map the olfactory-semantic space of the descriptors. This was done by categorizing the descriptors, on the one hand, and by applying a Principal Component Analysis (PCA) to establish the dimensions along which the descriptors are differentiated semantically, on the other. We finally selected 38 descriptors that are evenly dispersed across this olfactory-semantic space. This method is described in detail in Supplementary Materials Sect. 2. The resulting olfactory-semantic space, with the selected descriptors highlighted, is illustrated in Supplementary Fig. 3. The full list of descriptors together with their semantic properties can be found in Supplementary Table 2.

### Statistical analysis

All data were analyzed with the statistical software R [28]. Analyses investigated factors determining whether a particular odor was classified as being perceived qualitatively different (i.e., *Qualitatively different*), on the one hand, and whether it was perceived at all (i.e., *Odorless*), on the other. The factor Qualitatively different differentiated *qualitatively different-*responses from *odorless-* and *normal-*responses. The factor Odorless differentiated *odorless-*responses from *qualitatively different-* and *normal-*responses. *Unknown-*responses were categorized as missing data. We were interested in how *qualitatively different* and *odorless*-responses differ across the selected descriptors.

In particular, we asked whether parosmia responses are associated with the semantic properties of the descriptors. To this end, we investigated whether parosmia responses depended on any of the semantic variables OAI, OSI, Valence, Arousal, Gustatory association and Olfactory association. However, as shown in Table 1, some of these variables are correlated. Therefore, as described in more detail below, we conducted separate analyses for each semantic variable.

**Table 1:** Correlation matrix illustrating Pearson correlations between semantic variables

	OAI	OSI	Valence	Arousal	Olfactory	Gustatory
OAI	1.000	0.009	− 0.044	− 0.233	0.311	− 0.283
OSI	0.009	1.000	− 0.327	− 0.013	− 0.246	− 0.354
Valence	− 0.044	− 0.327	1.000	0.144	− 0.072	0.639
Arousal	-0.233	− 0.013	0.144	1.000	− 0.027	0.456
Olfactory	0.311	− 0.246	− 0.072	− 0.027	1.000	− 0.361
Gustatory	− 0.283	− 0.354	0.639	0.456	− 0.361	1.000

To further explore and control for factors related to the participants in the study, the following participant variables were also included in the main analyses: age, body mass index (BMI), sex, duration of olfactory impairment (parosmia or olfactory loss), olfactory-perceptual ability (TDI), experienced parosmia intensity, experienced parosmia valence, subjective impairment severity, and importance of olfaction. A few data points are missing for some of these variables. For subjective impairment severity, about 30% of the data are missing, and for BMI, 12.5% is missing. Less than 5% is missing for the rest of the variables (see Supplementary Table 3 for a complete list). These missing data points were imputed using the aregImpute function in the Hmisc package [9].^1^ Multiple additive regression models are fit on *n* bootstrap samples of the full data set. These models are then used to predict n vectors of missing data points of each of the variables. We used ten different bootstrap samples and used the mean of each vector as imputed values.

First, we asked if parosmic and anosmic sensations were associated with particular semantic features. We investigated the correlations between the semantic variables of each descriptor and the by-descriptor percentage of *qualitatively different*-responses, on the one hand, and *odorless*-responses, on the other, calculated across participants. The main analyses involved logistic mixed-effects modeling as implemented in the package lme4 [2]. These models predicted *qualitatively different*-responses, on the one hand, and *odorless*-responses, on the other. To deal with collinearity issues and to be able to assess the effect of each semantic variable on its own, without taking into account the influence of the other semantic variables, separate analyses were conducted for each semantic variable. Each of these analyses included participant variables listed above. In all of these models, all continuous variables were standardized. Following Gelman and Hill [7], standardization involved division by 2 standard deviations. The categorical variable Sex was effect-coded. All models also included a random participants-intercept.

We then derived the *parosmia severity index*, a measure of parosmia severity, on the basis of PCA on the semantic variables and the descriptor-level proportions of *qualitatively different*- and *odorless*-responses. Principal Component Analysis is a factorial method that describes and summarizes the variability and the relationships of a set of continuous variables, by showing how these can be described in terms of a smaller set of underlying, latent variables or principal components. The PCA analysis was performed with the PCA function in the FactoMineR package [17]. We use the resulting principal components to calculate the parosmia severity index, solely on the basis of the behavioral classification task, as described below.

## Results

### Semantic variables and impairment

**Fig. 1:**
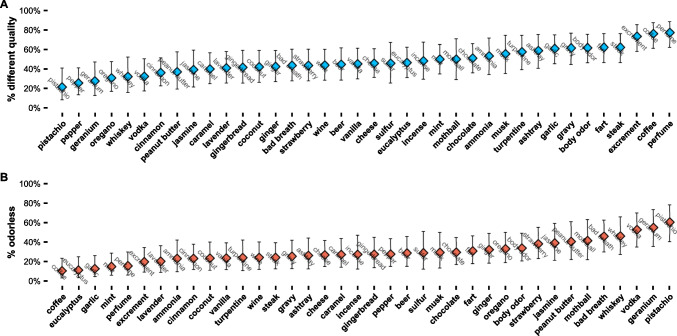
By-descriptor percentages, calculated across participants, of **A** *qualitatively different*- and **B** *odorless*-responses. Error bars illustrate 95% confidence intervals of these percentages, calculated on the basis of normal approximation

**Fig. 2:**
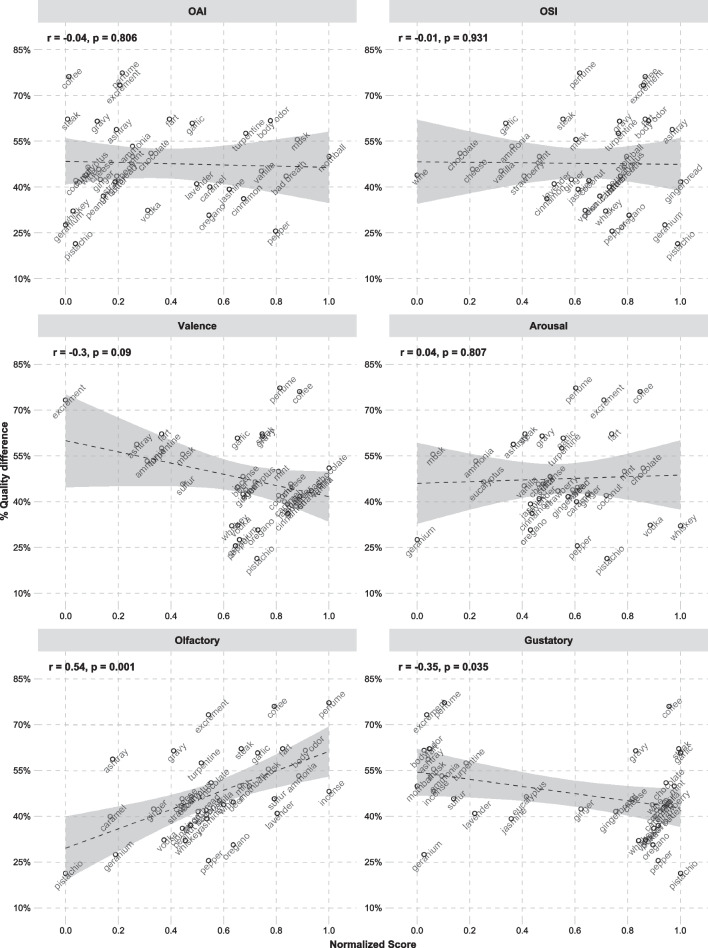
The relationships between semantic variables and percentage of *Qualitatively different*-responses, calculated across participants. Shaded areas illustrate 95% confidence intervals of the slopes

**Fig. 3:**
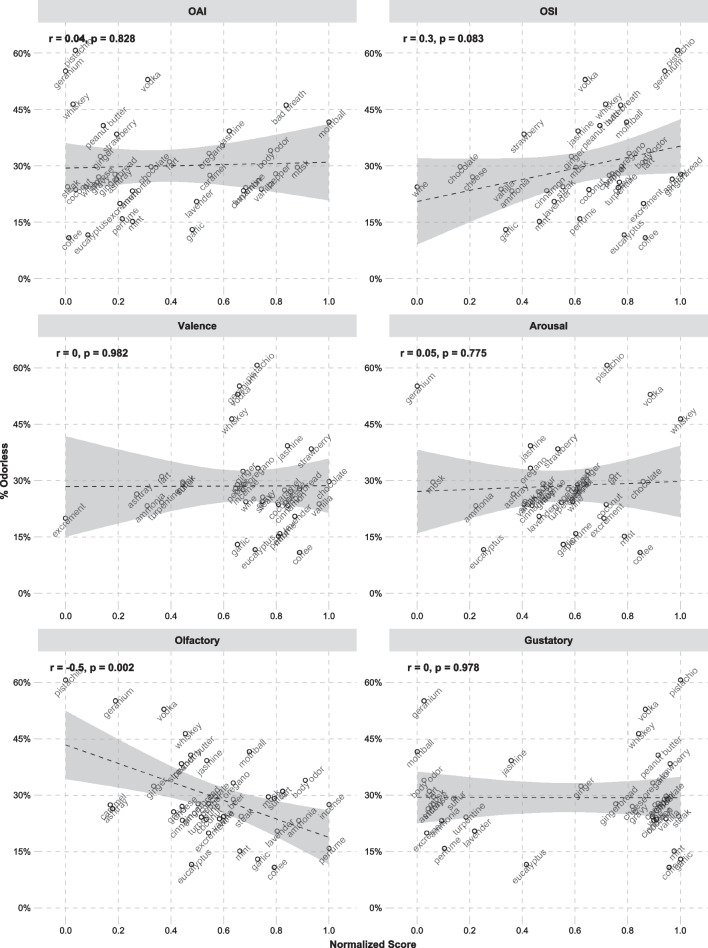
The relationships between semantic variables and percentage of *Odorless*-responses, calculated across participants. Shaded areas illustrate 95% confidence intervals of the slopes

For illustration, Fig. 1 shows the by-descriptor percentages of *qualitatively different*-responses and *odorless*-responses calculated across participants. The correlations between the semantic variables and the by-descriptor percentages of *qualitatively different*-responses and *odorless*-responses are shown in Table 2 and further illustrated in Figs. 2 and 3.

**Table 2:** Correlations between semantic variables and by-descriptor percentages of *qualitatively different*-responses and *odorless*-responses. Significant correlations are marked with a ʻ*ʼ

Variable	*n*	Qualitatively different		Odorless	
		*r*	*p*	r	*p*
OAI	35	− 0.04	0.807	0.04	0.828
OSI	35	− 0.02	0.932	0.30	0.083
Valence	34	− 0.30	0.090	0.00	0.982
Arousal	34	0.04	0.807	0.05	0.775
Olfactory	37	0.54	0.001*	− 0.50	0.002*
Gustatory	37	− 0.35	0.035*	− 0.01	0.978

The results of the logistic mixed-effects models predicting percentage of *qualitatively different*-responses are shown in Table 3 and the results of the models predicting percentage of *odorless*-responses are shown in Table 4. In both of these tables, the effects of the subject variables are taken from a model without any of the semantic variables, and the effects of the semantic variables come from each of the models including the semantic variable at hand. There were no qualitative differences in the effects of the subject-level variables in any of the models. Both the correlations and the logistic mixed-effects models show the same pattern of results for the linguistic variables. *Qualitatively different*-responses occur more frequently for descriptors that are more strongly associated with olfaction (e.g., “perfume”, “body odor”, “incense”) than for descriptors less related to olfaction (e.g., “pistachio”, “geranium”, “caramel”). This can be expected, since highly olfactory-related words probably describe smells that are highly noticeable and frequently experienced. *Qualitatively different*-responses are, interestingly, also most often reported for unpleasant odors from inedible sources (e.g., “excrement”, “ashtray”, “turpentine”)—whereas parosmic responses are *less* frequent for pleasant food odors (e.g., “strawberry”, “chocolate”, “wine”). *Odorless*-responses are in contrast more frequent for descriptors that are high in OSI, thereby being used in a limited set of olfactory contexts (e.g., “whiskey”, “mothball”, “gingerbread”). Such descriptors presumably denote more specific odor experiences in comparison to more general descriptors that are used in a broader range of contexts (e.g., “wine”, “cheese”, “vanilla”*)*. In contrast to *qualitatively different-*responses, *odorless-*responses are also reported less frequently for descriptors with a strong association to olfaction (e.g., “perfume”, “body odor”, “incense”).

**Table 3:** Results of logistic mixed-effects models predicting *qualitatively different*-responses as a function of the participant variables and the semantic variables. Participant variables show results of a model without any semantic variables included, and semantic variables show results of individual models with participant variables and each semantic variable included individually. Significant effects are marked with a ʻ*ʼ

Predictor	Estimate	Std. error	z value	*p*
Participant variables				
(Intercept)	0.12	0.26	0.45	0.652
Age	0.10	0.61	0.17	0.867
BMI	− 1.41	0.57	− 2.46	0.014*
Sex	− 0.83	0.56	− 1.47	0.142
Olfactory-perceptual ability (TDI)	0.33	0.68	0.49	0.621
Duration impairment	− 0.18	0.56	− 0.32	0.752
Degree parosmia	− 0.83	0.62	− 1.33	0.182
Intensity parosmia	1.84	0.70	2.62	0.009*
Valence parosmia	− 0.16	0.65	− 0.24	0.808
Subjective impairment severity	− 0.16	0.62	− 0.26	0.797
Importance of olfaction	0.23	0.67	0.34	0.730
Semantic variables				
OAI	− 0.18	0.14	− 1.29	0.197
OSI	0.16	0.13	1.23	0.217
Valence	− 0.59	0.14	− 4.21	< 0.001*
Arousal	0.12	0.15	0.83	0.408
Olfactory	0.95	0.15	6.46	< 0.001*
Gustatory	− 0.79	0.14	− 5.58	< 0.001*

**Table 4:** Results of logistic mixed-effects models predicting *odorless*-responses as a function of the participant variables and the semantic variables

Predictor	Estimate	Std. error	z value	*p*
Participant variables				
(Intercept)	− 1.90	0.33	− 5.67	< 0.001*
Age	− 0.13	0.76	− 0.17	0.866
BMI	1.88	0.75	2.51	0.012*
Sex	− 0.64	0.70	− 0.91	0.363
Olfactory-perceptual ability (TDI)	− 3.13	0.86	− 3.65	< 0.001*
Duration impairment	− 0.14	0.70	− 0.20	0.840
Degree parosmia	1.26	0.79	1.60	0.110
Experienced intensity	− 1.66	0.90	− 1.85	0.065
Experienced valence	1.39	0.81	1.72	0.085
Subjective impairment severity	0.62	0.79	0.78	0.436
Importance of olfaction	1.02	0.82	1.24	0.215
Semantic variables				
OAI	0.22	0.18	1.19	0.236
OSI	0.41	0.18	2.32	0.020*
Valence	0.17	0.19	0.94	0.348
Arousal	0.23	0.19	1.19	0.236
Olfactory	− 1.03	0.19	− 5.28	< 0.001*
Gustatory	0.18	0.18	0.97	0.331

Participant variables show results of a model without any semantic variables included, and semantic variables show results of individual models with participant variables and each semantic variable included individually. Significant effects are marked with a ‘*’

We leveraged participant variables to understand individual differences in parosmia and anosmia (Table 3). Our analyses show that a higher BMI predicts more frequent *odorless*-responses, but less frequent *qualitatively different-*responses, even when age is controlled for. As perhaps can be expected, the intensity of parosmia experiences also predicts more frequent *qualitatively different*-responses, and a higher olfactory-perceptual ability (TDI) predicts fewer *odorless-*responses.

### Parosmia severity index

On the basis of PCA, we finally derived a parosmia severity index (an analysis and interpretation of the three first principal components is presented in Supplementary Materials Sect. 4). We reasoned that odors that are less likely to be affected by parosmia are indicative of greater parosmia severity, as those are the ones that should be affected last. We thus calculated a severity score that is based on the number of affected odors that are classified as *qualitatively different* (*QD*) or *odorless* (*OL*). Importantly, the influence of each odor is weighed with respect to how strongly it is associated with these affectedness-responses, so that those odors that are the least often affected are weighted more strongly. The weights are calculated on the basis of the PCA model in the following way. First, weighted PC scores *OLs* and *QDs* are obtained by multiplying the scores *s* on principal component *p* with their Pearson correlations to the odorless *ol* and qualitatively different *qd* percentages of descriptors *d*

$${\mathrm{OLs}}_{p, d}={{s}_{p,d}\rho }_{{s}_{p},ol}$$

$${\mathrm{QDs}}_{p, d}={{s}_{p,d}\rho }_{{s}_{p},qd}$$

OL and QD weights for descriptor *d* are then obtained by weighing the inverses of the normalized scores *OLs* and *QDs* by the percentage of variance *v* that is explained by PC *p*, and summing across PCs

$${\mathrm{OL}w}_{d}={\sum }_{d}\left(1 -\frac{{\mathrm{OL}s}_{p,d}-{\mathrm{OL}s}_{\mathrm{min}}}{{\mathrm{OL}s}_{\mathrm{max}}-{\mathrm{OL}s}_{\mathrm{min}}}\right){v}_{p}$$

$${\mathrm{QD}w}_{d}={\sum }_{d}\left(1 -\frac{{\mathrm{QD}s}_{p,d}-{\mathrm{QD}s}_{\mathrm{min}}}{{\mathrm{QD}s}_{\mathrm{max}}-{\mathrm{QD}s}_{\mathrm{min}}}\right){v}_{p}$$

The strength of each odor-specific weight thus depends on how strongly each PC is related to *odorless* and *qualitatively different*-responses, on the one hand, and to their overall strength, on the other. A parosmia severity score *ps* for the *i*th participant is then calculated as

$${ps}_{i}=\frac{\sum {\mathrm{OL}}_{d, d\in {D}_{i}}{\mathrm{OL}w}_{d, d\in {D}_{i}},{\mathrm{QD}}_{d, d\in {D}_{i}}{\mathrm{QD}w}_{d, d\in {D}_{i}}}{\sum {\underset{d\in {D}_{i}}{\mathrm{max}}\left\{{\mathrm{OL}w}_{d},{\mathrm{QD}w}_{d}\right\}}_{d}},$$

where *D* is the set of descriptors that have been classified by participant *i*, and *OL* and *QD* are vectors of *odorless* or *qualitatively different-*responses, coded as 1 or 0, provided by participant *i* on *d* in *D*.

**Fig. 4:**
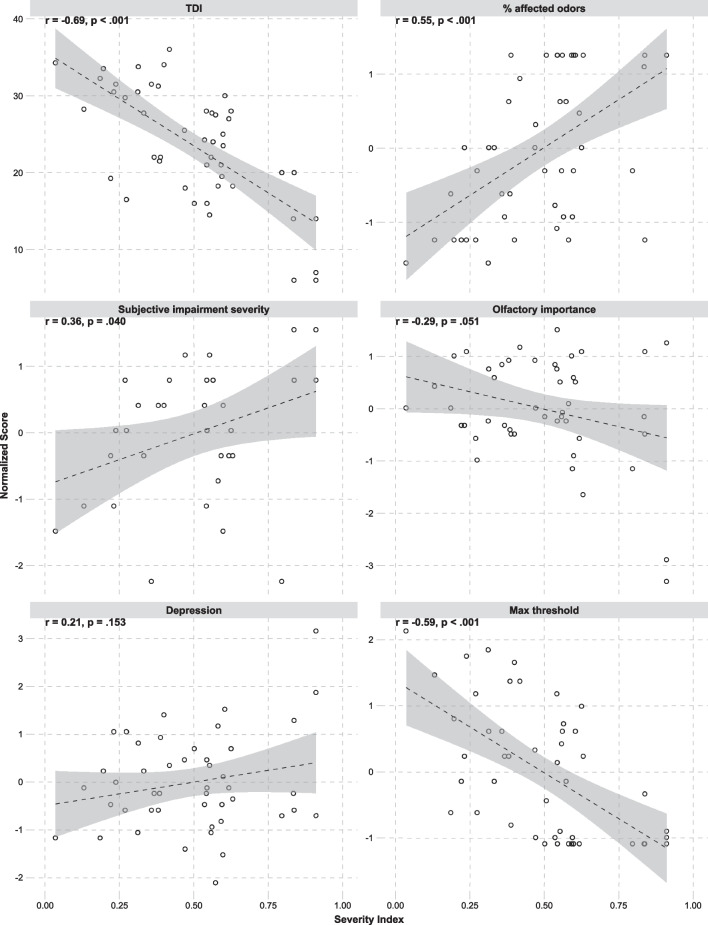
The relationships between the parosmia severity index and some of the participant variables. Shaded areas illustrate 95% confidence intervals of the slopes

We evaluated our parosmia severity index against several of the participant variables. Results show that the severity index is positively related to the percentage of odors reported as affected, subjective impairment severity, and to a small extent also to depression. It is negatively related to olfactory-perceptual ability (TDI score), maximum nasal threshold, and to some extent to the self-rated olfactory importance. These relationships are illustrated in Fig. 4.

## Discussion

Parosmia is increasingly regarded as a condition that might vary widely among affected individuals [18, 23]. New quantitative methods are needed to characterize the varying nature and impact of parosmia. The odor sources that are associated with parosmic sensations, as well as those that fail to elicit any olfactory sensations in parosmic individuals, are not well understood. Here, we used a new methodology that is based on the linguistic study of semantics and that uses written natural language to characterize the odor terms that are often reported in parosmia and smell loss. With that methodology, we were able to make inferences about the organization of parosmic and anosmic sensations. Our new tool may facilitate the comparisons of parosmia severity and its changes over time, and the assessment has a survey format that makes it easy to administer.

We found that the parosmic experiences are most commonly reported for odor terms that describe odors that are known to be unpleasant and not related to food (e.g., “excrement”), and that are more strongly associated with olfaction. These findings differ to some extent from those of, e.g., Parker et al. [25] and Pellegrino et al. [27] who found parosmic experiences to most often be triggered by roasted, fried, or sulfurous food products (e.g., coffee, garlic, bacon, and toast). Although some of these products are highlighted as common parosmia triggers also in our data (i.e., coffee and garlic), our results suggest that, in general, unpleasant odors unrelated to food more frequently engender parosmic experiences. We suggest that pleasant food odors are so commonly reported in parosmia, because they are often encountered and their distortions have more severe consequences for everyday life. Parosmic patients may therefore be biased toward remembering and reporting such odors. Since our method uses a pre-determined set of terms, it should be less affected by such bias. We note, however, that our participants were more inclined to report parosmic experiences for odor terms more strongly associated with olfaction. They might thus have been biased toward reporting odors that are highly noticeable and frequently experienced. In sum, our findings provide new insights about what type of odors that most commonly are parosmic, and indicate that parosmic patients might be biased in their self-reports. Presenting patients with a pre-determined list of odor descriptors, as in our method, might help assess and control the effects of this bias.

On the basis of PCA modeling of the semantic properties of the odor descriptors together with the behavioral data of odor classifications, we then derived the parosmia severity index, a measure of parosmia severity that can be determined solely from the behavioral odor categorisation task. This parosmia severity score appears promising, because it is associated with general olfactory-perceptual ability (TDI), the percentage of odors reported as affected, subjective impairment severity, depression, and olfactory sensitivity. The interest comes from the fact that in the clinical field so far, patients are only asked for presence or absence of parosmia [11]. This approach is problematic, because the lack of a quantitative, gradual measure prohibits the tracking of changes during the course of the olfactory disorder. It also makes it difficult to evaluate potential therapeutic effects. There are many measures of quantitative olfactory loss [8]. But only recently has a quantitative measure of parosmia been published, the SSParoT, which uses olfactory stimulation [18]. However, this test has not yet been fully validated. Furthermore, there is a risk that patients might resist parosmia assessments that involve olfactory exposure due to highly unpleasant odor distortions. Our proposed quantitative measure of parosmia severity might therefore be highly valuable in a clinical setting as it can be used to track the development of the disorder over time, and that it does not require exposure to actual odors. Our measure awaits further validation in a prospective clinical studies.

In terms of individual differences, we found that neither age nor sex affected the responses. Parosmias have been reported to be more frequently present in younger individuals, and in participants with better olfactory function [27], but a recent population-based study on parosmic sensations showed no association with age or sex [23]. High BMI was associated with more anosmic, but fewer parosmic sensations. This finding is in line with observations, suggesting that whereas individuals with parosmias tend to reduce their food intake due to loss of appetite, those with anosmias increase their intake of high-carbon and high-fat foods containing more sugar and salt, to compensate for their loss of flavor [3], see also [1]. More research is needed to confirm whether this association is driven by changes in food behaviors.

In sum, we present a data-driven approach to understanding distorted smell sensations, parosmia. Our method reveals which odors are most common triggers of parosmia. We developed a parosmia severity index that appears to capture meaningful variation among patients in terms of the extent and impact of their parosmia. This measure can be used to track changes in the development of disorder over time and does not require odor exposure that potentially triggers highly unpleasant distortions. Future research should further validate this method as a quantitative research tool to understand parosmia, which is a common complaint but which has yet been elusive, because of its qualitative nature and its variability across patients and over time.

## Supplementary Information

Below is the link to the electronic supplementary material.

Supplementary file1 (PDF 3469 KB)

## Data Availability

The data underlying this article are available in the Open Science Framework (OSF) Repository, at https://osf.io/ejqbr/.

## Footnotes

1 Importantly, additional analyses found that the effects identified below also hold without these imputations.
