# Supplementary material for: A parosmia severity index based on word-classification predicts olfactory abilities and impairment
Source: Eur Arch Otorhinolaryngol. 2023 Mar 11;280(8):3695–706. doi: 10.1007/s00405-023-07893-2 (PMC10008075; doi:10.1007/s00405-023-07893-2)
Supplement: Supplementary file 1 — Supplementary file1 (PDF 3469 KB) [file 405_2023_7893_MOESM1_ESM.pdf]

A parosmia severity index based on word classification predicts olfactory abilities and impairment - Supplements

Thomas Hörberg[1*], Rumi Sekine[2, 3], Clara Overbeck[2], Thomas Hummel[2] and Jonas K. Olofsson[1]

[1]Department of Psychology, Stockholm University, Sweden

[2]Smell & Taste Clinic, Department of Otorhinolaryngology, TU Dresden, Germany

[3]Department of Otorhinolaryngology, Jikei School of Medicine, Tokyo, Japan

1. Corpus data

All corpus-based analyses were done using the University of Maryland Baltimore County (UMBC) webbase corpus (Han et al., 2013). This corpus consists of English written texts of more than three billion words in 100 million web pages from over 50,000 websites. The preprocessing of the corpus was done as described in Hörberg et al. (2020). The olfactory sub-corpus, consisting of 7.9 million words in 293 960 olfactory and gustatory contexts (also described in more detail in Hörberg et al. 2020) was also used. These contexts were identified on the basis of 105 olfactory- or gustatory-related key words (e.g., ’odor’, ’fragrance’, ’taste’, ’savor’, see Hörberg et al., 2020).

2. Descriptor identification

Following Hörberg et al. (2020), we extracted all nouns occurring at least 20 times within a ±4 word olfactory/gustatory context window of the olfactory/gustatory key words. For these nouns, we calculated Olfactory Association Index (OAI) and Olfactory Specificity Index (OSI), removed unsuitable terms, and extracted their lemmas (Hörberg et al., 2020). We then selected the top 200 nouns with the highest olfactory association in terms of OAI. The semantic distances between these nouns were calculated on the basis of word embeddings from a distributional-semantic word embedding model that was trained on our olfactory sub-corpus. Word embeddings represent semantic distances between words as vector distances in a multi-dimensional space. Our model was trained with the continuous-bag-of-words (CBOW) algorithm of word2vec (Mikolov et al., 2013), using the same parameters as in Hörberg et. al (2020). For each descriptor word embedding pair $\{i, j\}$, the distance D was calculated as

$$D_{i,j} = 0.5 \times (1 - \rho_{i,j})$$

The distance D is thus the pearson (ρ) correlation between word embeddings, converted to the 0-1 range, 0 reflecting semantic identity (i.e., corresponding to $\rho = 1$) and 1 indicating semantic opposition (i.e., corresponding to $\rho = -1$).

The resulting distances were used to categorize the descriptors into semantic categories, on the one hand, and to derive the dimensions along which the descriptors are differentiated semantically, on the other. Semantic clusters were identified using Agglomerative Nesting clustering (AGNES, see Kaufman & Rousseeuw, 1990) as implemented in the R package 'Cluster' (Maechler et al., 2019). From this clustering, we divived the descriptors into seven different semantic clusters (named *Stale & Chemical*, *Refreshing*, *Flowery & Fragrant*, *Edible*, *Fruity & Sweet*, *Alcoholic*, and *Herbal & Spicy*). This was done on the basis of our inspection and interpretation of the data (an illustration of the clustering is shown in Supplementary Figure 1). The primary semantic dimensions of the descriptors were derived with principal component analysis (PCA), as implemented in the prcomp() function the base R stats package (R Core Development Team, 2018). Following Hörberg et al. (2020), we focused on the first three principal components, explaining a total of 64.80% (PC1: 38.33%, PC2: 18.30%, PC3: 8.17%) of the variance of the data. In order to interpret these semantic dimensions, we

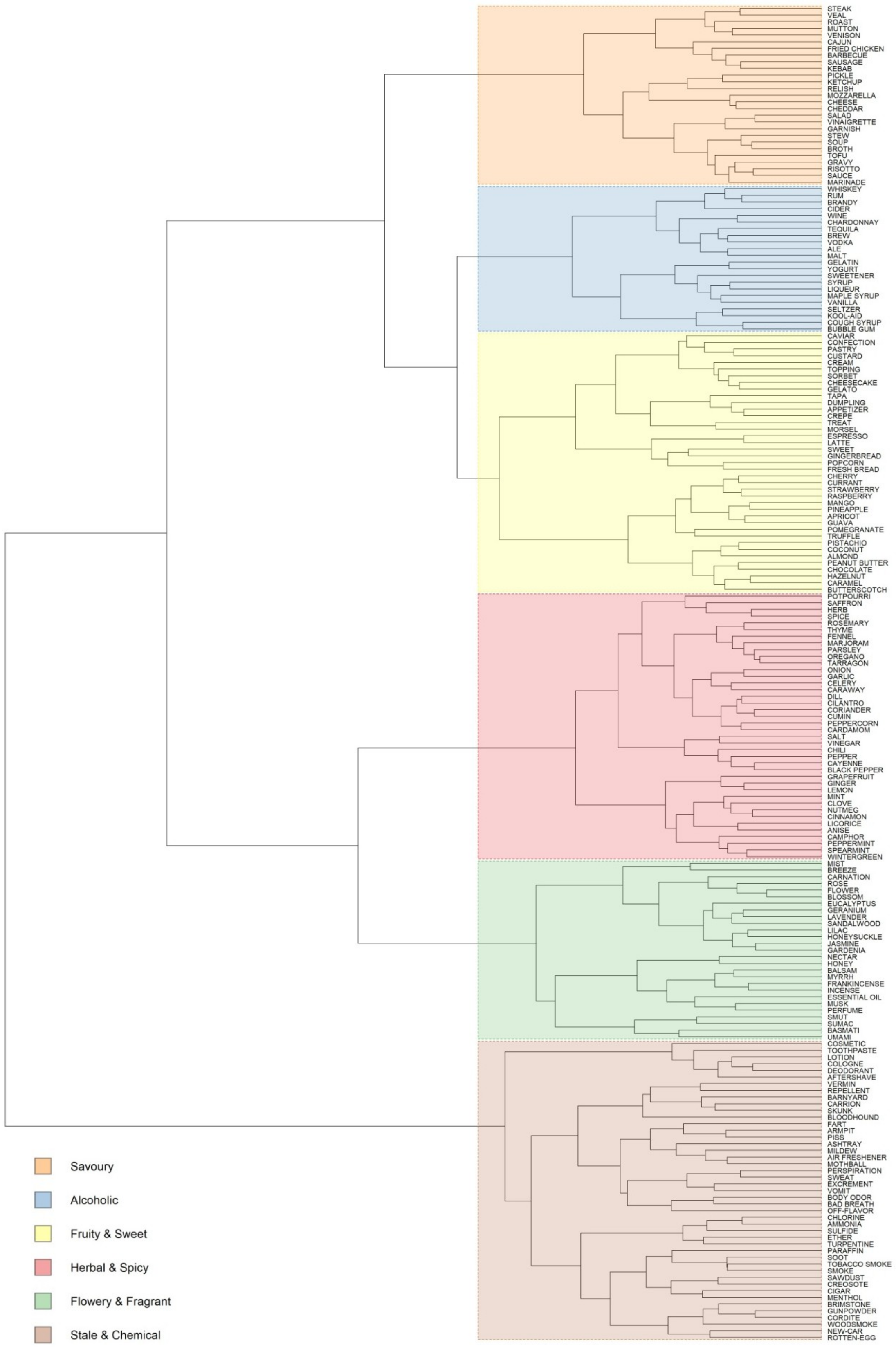

Supplementary Figure 1. Hierarchical clustering of the top 200 descriptors.

investigated their correlations with the lexical-semantic variables (OAI, OSI, Descriptor frequency, Valence, Arousal, Olfactory and Gustatory associations). These correlations are shown in Supplementary Table 1. Supplementary Figure 2 illustrates the loadings and contributions of the top 40 and bottom 40 descriptors of each PC component.

Supplementary Table 1. Correlations between the first three principal components of the PCA model of the top 200 descriptors, on the one hand, and the lexical-semantic variables, on the other. P-values are corrected for multiple comparisons using the method of Holm (1979). Shadings highlight significant correlations.

**Lexical-semantic variable**	**PC1**		**PC2**		**PC3**	
	r	*p*	*r*	*p*	*r*	*p*
OAI	-0.19	.058	0.32	<.001	-0.02	1.00
OSI	-0.03	1.00	-0.09	.570	-0.00	1.00
Frequency	0.12	.437	-0.09	.570	0.01	1.00
Valence	0.51	<.001	0.09	.570	-0.04	1.00
Arousal	-0.11	.702	-0.18	.174	-0.16	.397
Gustation	0.80	<.001	-0.15	.274	-0.15	.397
Olfaction	-0.02	1.00	0.33	<.001	0.06	1.00
Concreteness	0.20	.058	0.13	.374	-0.08	1.00
Imageability	0.12	1.00	0.36	.374	0.10	1.00

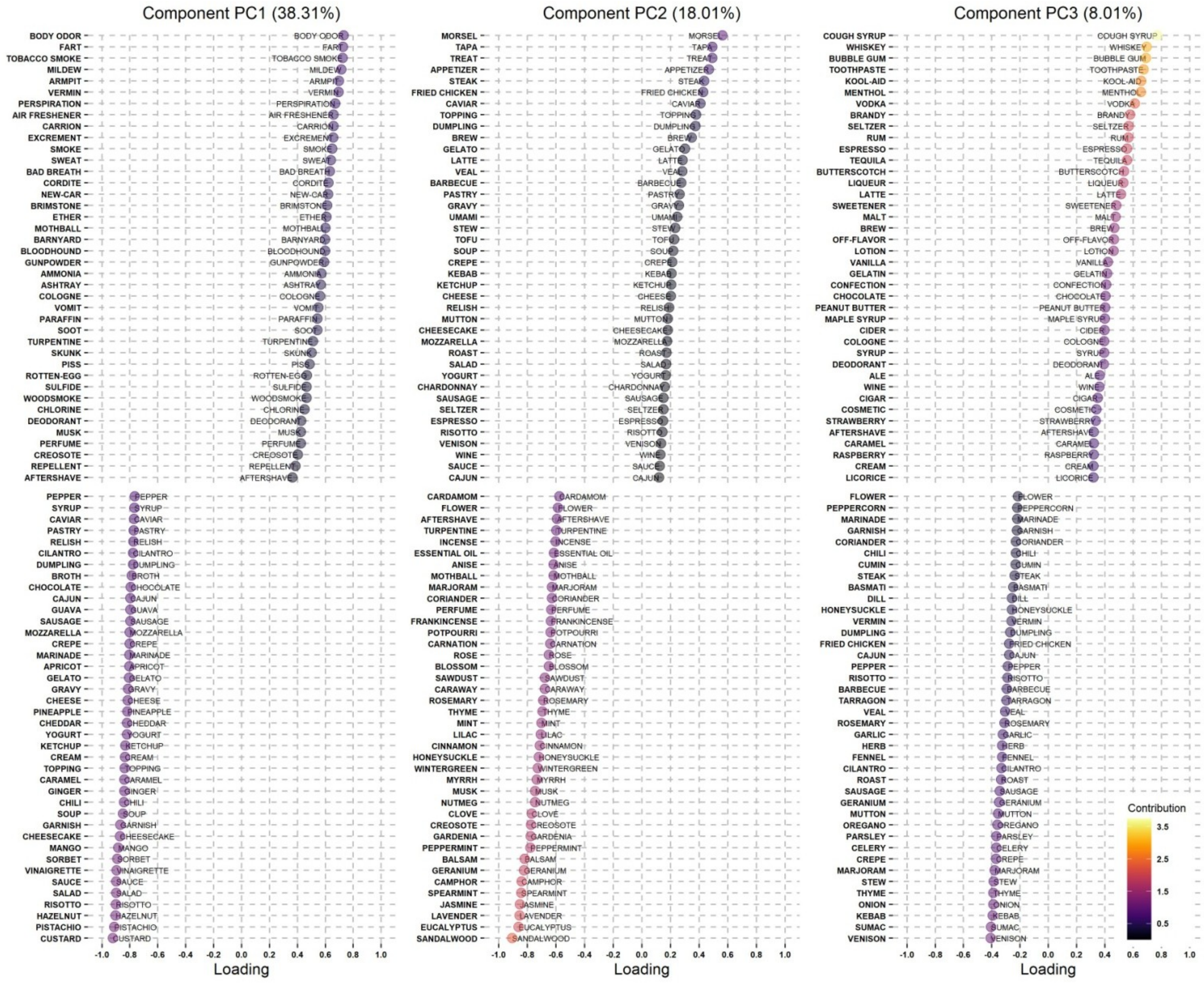

Supplementary Figure 2. Loadings and contributions of the top 40 and bottom 40 descriptors of each component

As PC1 is highly correlated with both gustatory association ratings (r = 0.80, p < .0001) and word valence ratings (r = 0.51, p < .0001), it primarily seems to differentiate between descriptors referring to edibles with pleasant smells (e.g., *custard, mango, caramel*) and those referring to unpleasant inedibles (e.g., *fart, armpit, bad breath*). PC2, on the other hand, is unrelated to both edibility and valence, showing no correlations to neither gustatory association ratings nor word valence ratings. Instead, PC2 is correlated with OAI (r = 0.32, p < .0001) and olfactory association ratings (r = 0.33, p < .0001), indicating that is related to olfactory association strength. As such, PC2 differentiates between descriptors that are strongly associated to olfaction (e.g., *lavender, musk, perfume*) from those with a weaker olfactory association (e.g., *steak, caviar, espresso*). PC3 is not correlated to any of the lexical-semantic variables, but seems to differentiate alcoholic (e.g., *vodka, whiskey, tequila*), sweet (e.g., *vanilla, maple syrup, caramel*), minty (*cough syrup*, *menthol, toothpaste*) and fragrant (*lotion*, *deodorant, cologne*) sources from more heavy, salty and savory edibles (e.g., *venison, stew*, *garlic*), possibly differentiating more light from more heavy odor sources. The semantic space of the 200 descriptors derived with the clustering and the PCA analysis is illustrated in Supplementary Figure 3. The figure illustrates the descriptors with respect to their distribution on the principal components, on the one hand, and with regard to their semantic clustering, on the other. The full list of descriptors together with their semantic properties can be found in Supplementary Table 2 below.

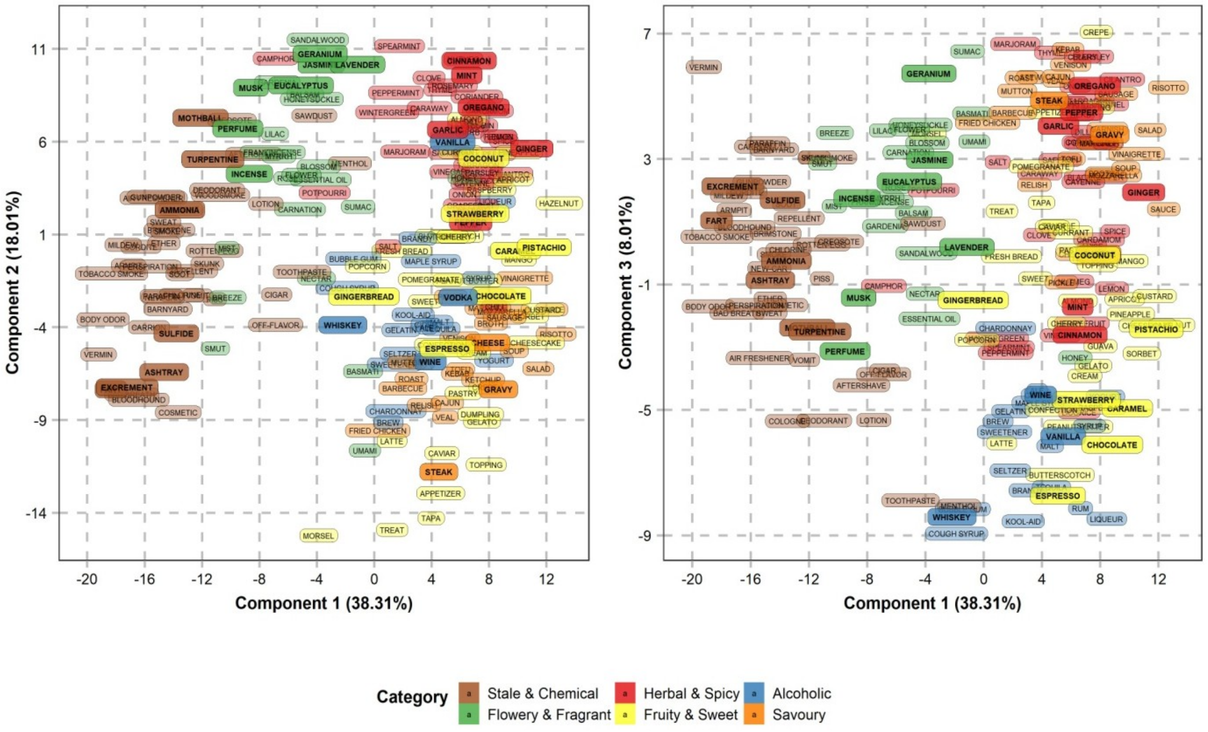

Supplementary Figure 3. The semantic space of the top 200 descriptors, in terms of PCA scores and clusterings. Left panel: PC1 and PC2 scores. Right panel: PC1 and PC3 scores. The descriptors that were selected to probe for perceptual distortions are illustrated in bold face.

Supplementary Table 2. Semantic properties, semantic category and PC scores for the top 200 descriptors.

Descriptor	OAI	OSI	Freq.	Arousal	Valence	Olf. ass.	Gust. ass.	Category	PC1	PC2	PC3
ROTTEN-EGG	4.56	0.85	-3.00	0.99	-2.53	0.67	-0.99	Stale & Chemical	-11.138	0.135	0.281
WOODSMOKE	3.65	0.92	-2.58	-0.65	0.07	-0.89	-1.40	Stale & Chemical	-10.717	3.135	3.053
CORDITE	3.43	0.64	-2.25	NA	NA	NA	NA	Stale & Chemical	-16.269	0.337	1.947
UMAMI	3.31	0.96	-2.44	NA	NA	NA	NA	Flowery & Fragrant	-0.657	-10.628	3.559
OFF-FLAVOR	2.74	0.85	-2.06	NA	NA	NA	NA	Stale & Chemical	-6.878	-3.857	-3.860
AFTERSHAVE	2.26	0.92	-1.87	-0.65	-0.15	0.94	-1.58	Stale & Chemical	-8.401	5.338	-4.206
WINTERGREEN	2.17	0.42	-1.43	NA	NA	0.40	0.23	Herbal & Spicy	0.901	7.592	-2.707
GARDENIA	1.99	0.56	-1.45	-0.37	1.25	0.31	-1.12	Flowery & Fragrant	-6.767	9.205	0.863
MOTHBALL	1.91	0.31	-1.22	-1.93	-1.04	1.11	-1.58	Stale & Chemical	-12.115	7.289	-2.378
ANISE	1.76	0.14	-1.01	NA	NA	NA	NA	Herbal & Spicy	7.450	7.897	0.220
BUTTERSCOTCH	1.74	1.00	-1.66	-0.68	0.67	0.27	0.82	Fruity & Sweet	5.234	0.932	-7.063
NEW-CAR	1.65	1.04	-1.65	NA	NA	NA	NA	Stale & Chemical	-14.718	-2.385	-0.479
SPEARMINT	1.65	1.17	-1.75	0.96	-1.07	1.11	1.08	Herbal & Spicy	1.752	11.163	-2.994
CAMPHOR	1.58	0.22	-0.98	NA	NA	NA	NA	Herbal & Spicy	-6.872	10.499	-1.044
PERFUME	1.58	-2.25	0.92	0.40	0.70	2.14	-1.30	Flowery & Fragrant	-9.515	6.716	-3.119
MUSK	1.55	-0.49	-0.42	-2.07	-1.01	1.36	-1.41	Flowery & Fragrant	-8.599	8.909	-1.415
FRESH BREAD	1.51	0.75	-1.36	NA	NA	NA	NA	Fruity & Sweet	1.904	0.115	-0.099
BAD BREATH	1.42	0.22	-0.90	NA	NA	NA	NA	Stale & Chemical	-16.924	-7.605	-1.907
LICORICE	1.40	0.07	-0.78	0.21	-0.95	-0.33	0.90	Herbal & Spicy	6.550	6.901	-5.073
BLACK.PEPPER	1.30	0.09	-0.74	NA	NA	NA	NA	Herbal & Spicy	7.682	5.351	2.423
BODY.ODOR	1.24	0.66	-1.15	0.85	0.18	-0.43	-1.13	Stale & Chemical	-18.904	-3.555	-1.698
INCENSE	1.21	-1.57	0.58	-0.05	0.05	2.14	-1.41	Flowery & Fragrant	-8.704	4.283	1.757
AIR.FRESHENER	1.20	0.75	-1.19	-0.99	0.80	-0.95	-1.37	Stale & Chemical	-15.387	2.975	-3.339
CARDAMOM	1.19	0.61	-1.08	0.07	0.74	-1.28	-1.58	Herbal & Spicy	7.913	7.666	0.442
MORSEL	1.18	-0.85	0.05	-0.78	-0.36	-1.00	0.10	Fruity & Sweet	-3.871	-15.215	3.819
BUBBLE.GUM	1.17	0.88	-1.28	0.28	0.57	-2.23	-1.18	Alcoholic	-1.451	-0.301	-8.158
VANILLA	1.14	-1.67	0.70	-0.49	1.39	0.73	0.98	Alcoholic	5.460	5.993	-5.835
TARRAGON	1.11	0.88	-1.25	-0.45	-1.51	0.32	-1.47	Herbal & Spicy	7.264	4.748	4.850
MARJORAM	1.09	1.04	-1.36	NA	NA	NA	NA	Herbal & Spicy	2.163	5.465	6.670
CARAWAY	1.09	0.96	-1.30	0.07	0.74	-1.28	-1.58	Herbal & Spicy	3.845	7.814	2.542
THYME	1.03	-0.47	-0.17	NA	NA	0.13	0.40	Herbal & Spicy	4.595	8.804	6.397
FRANKINCENSE	1.01	0.56	-0.94	NA	NA	1.49	-1.48	Flowery & Fragrant	-7.082	5.436	1.632
MYRRH	0.99	0.31	-0.75	NA	NA	NA	NA	Flowery & Fragrant	-6.650	5.233	1.762
BRIMSTONE	0.96	0.15	-0.61	-0.16	-1.70	-1.05	-1.50	Stale & Chemical	-14.281	1.260	0.625
TURPENTINE	0.96	0.19	-0.64	0.15	-1.38	0.49	-1.08	Stale & Chemical	-11.259	5.061	-2.500
CINNAMON	0.93	-0.95	0.25	-0.36	0.84	0.25	0.85	Herbal & Spicy	6.606	10.379	-2.605
SANDALWOOD	0.88	0.56	-0.88	NA	NA	-0.68	-1.58	Flowery & Fragrant	-3.939	11.496	0.001
ESSENTIAL OIL	0.77	-0.46	-0.04	NA	NA	NA	NA	Flowery & Fragrant	-3.729	3.961	-2.083
JASMINE	0.77	-0.45	-0.05	-0.38	0.83	0.56	-0.61	Flowery & Fragrant	-3.712	10.157	2.979
MENTHOL	0.76	1.08	-1.22	-1.46	-0.68	0.18	0.30	Stale & Chemical	-1.704	4.843	-8.056
PEPPERCORN	0.73	1.12	-1.24	0.42	-0.08	0.53	0.69	Herbal & Spicy	7.552	3.914	3.515
HONEYSUCKLE	0.71	0.12	-0.46	0.53	1.26	1.11	0.08	Flowery & Fragrant	-4.351	8.300	4.091
LILAC	0.64	-0.06	-0.28	-0.47	1.00	0.61	-1.44	Flowery & Fragrant	-6.934	6.440	3.912
PEPPERMINT	0.63	0.64	-0.81	0.72	0.91	0.80	0.88	Herbal & Spicy	1.507	8.653	-3.171
GELATO	0.60	0.58	-0.75	-1.04	-0.05	-2.05	-1.36	Fruity & Sweet	7.514	-9.073	-3.579
SORBET	0.56	0.88	-0.97	NA	NA	-0.91	0.90	Fruity & Sweet	10.843	-3.400	-3.196
LIQUEUR	0.55	0.42	-0.60	1.43	-0.82	-0.02	0.93	Alcoholic	8.435	2.789	-8.469
CARAMEL	0.55	0.04	-0.31	0.41	0.78	-0.68	0.86	Fruity & Sweet	9.855	0.129	-4.942
CUMIN	0.55	0.56	-0.71	NA	NA	1.16	0.88	Herbal & Spicy	7.510	6.877	3.506
SPICE	0.54	-2.16	1.38	0.10	0.67	1.16	1.07	Herbal & Spicy	9.028	6.042	0.707
OREGANO	0.54	0.35	-0.55	-0.38	0.31	0.90	0.84	Herbal & Spicy	7.626	7.863	5.337
BLOODHOUND	0.52	0.85	-0.92	0.81	-0.60	-1.48	-1.48	Stale & Chemical	-16.414	-7.898	0.837
SUMAC	0.50	0.88	-0.94	NA	NA	NA	NA	Flowery & Fragrant	-1.124	2.508	6.435
BASMATI	0.50	1.17	-1.15	NA	NA	NA	NA	Flowery & Fragrant	-0.793	-6.373	4.455
NUTMEG	0.48	0.20	-0.40	-1.00	0.02	0.01	0.37	Herbal & Spicy	6.203	10.462	-0.898
CORIANDER	0.48	0.72	-0.80	NA	NA	NA	NA	Herbal & Spicy	7.150	8.431	3.540
COUGH SYRUP	0.46	1.12	-1.10	0.69	-1.78	-1.83	-1.02	Alcoholic	-1.883	-1.882	-8.933
FENNEL	0.42	0.20	-0.37	-0.53	-0.31	NA	NA	Herbal & Spicy	8.682	6.291	4.749
LAVENDER	0.40	-0.83	0.44	-0.24	0.96	1.47	-0.99	Flowery & Fragrant	-1.160	10.170	0.198
TRUFFLE	0.38	0.12	-0.29	-0.65	0.80	0.29	0.58	Fruity & Sweet	5.166	0.877	0.876
GARLIC	0.35	-1.60	1.05	0.18	-0.05	1.22	1.13	Herbal & Spicy	5.134	6.657	4.058
VINEGAR	0.34	-1.34	0.85	-0.59	-0.33	1.68	1.00	Herbal & Spicy	5.264	4.392	-2.620
CILANTRO	0.31	0.27	-0.37	-1.40	0.06	0.00	0.56	Herbal & Spicy	9.516	4.273	5.546
PEPPER	0.31	-2.40	1.69	0.42	-0.08	0.59	0.90	Herbal & Spicy	6.718	1.677	4.500

CLOVE	0.28	-0.11	-0.06	-0.90	-0.17	1.09	0.27	Herbal & Spicy	3.821	9.453	0.581
CREPE	0.27	0.56	-0.56	-0.49	0.61	-0.73	0.25	Fruity & Sweet	7.729	-7.225	7.052
PISS	0.21	1.17	-1.01	1.02	-2.47	0.60	-1.40	Stale & Chemical	-11.109	-2.483	-0.804
MARINADE	0.20	0.42	-0.42	NA	NA	0.18	0.72	Savoury	7.916	-2.950	3.501
GUNPOWDER	0.20	-0.28	0.11	1.64	-0.30	0.82	-1.35	Stale & Chemical	-15.232	3.037	2.283
SKUNK	0.17	-0.55	0.34	2.46	-1.60	1.28	-1.51	Stale & Chemical	-11.644	-0.564	3.048
DEODORANT	0.16	0.37	-0.37	-0.34	0.29	1.37	-1.34	Stale & Chemical	-10.958	3.417	-5.328
MAPLE SYRUP	0.16	0.42	-0.40	-0.29	0.29	-0.45	-0.20	Alcoholic	3.960	-0.430	-4.771
POTPOURRI	0.15	0.75	-0.66	-1.41	-0.19	1.59	-1.52	Herbal & Spicy	-3.464	3.255	2.014
VOMIT	0.12	0.29	-0.29	1.12	-3.08	0.62	0.10	Stale & Chemical	-12.315	-2.279	-3.404
CARRION	0.10	0.56	-0.48	0.07	0.74	-1.28	-1.58	Stale & Chemical	-15.700	-3.992	3.404
FART	0.09	0.61	-0.51	1.02	-1.38	1.54	-1.45	Stale & Chemical	-18.355	-7.340	1.045
SMOKE	0.05	-3.17	2.41	1.37	-1.88	1.53	-0.75	Stale & Chemical	-14.427	1.183	-0.040
SAUCE	0.01	-2.21	1.69	-0.05	0.70	0.82	0.86	Savoury	12.354	-3.032	1.407
BROTH	0.00	-0.68	0.52	-2.31	-0.15	0.23	0.80	Savoury	8.124	-3.780	2.545
STEW	-0.02	-0.77	0.60	-1.40	0.10	1.70	1.09	Savoury	3.226	-5.890	5.619
SAFFRON	-0.02	0.27	-0.20	-1.33	0.36	0.60	0.06	Herbal & Spicy	5.265	5.403	2.976
MALT	-0.04	-0.13	0.12	-0.32	0.33	-0.79	0.78	Alcoholic	4.562	-3.711	-6.142
ARMPIT	-0.07	0.33	-0.22	-0.56	-1.34	0.54	-1.46	Stale & Chemical	-17.135	-0.658	1.377
TOPPING	-0.07	0.72	-0.52	NA	NA	-2.40	-0.69	Fruity & Sweet	7.793	-11.404	-0.376
GUAVA	-0.08	0.96	-0.70	NA	NA	-0.04	0.76	Fruity & Sweet	8.010	2.155	-2.962
RASPBERRY	-0.09	-0.68	0.57	-0.37	1.29	0.08	1.05	Fruity & Sweet	8.035	3.420	-4.914
LEMON	-0.10	-1.19	0.96	0.72	0.52	0.14	0.92	Herbal & Spicy	8.795	6.283	-1.126
CHOCOLATE	-0.13	-2.33	1.85	1.56	1.56	0.62	0.98	Fruity & Sweet	8.821	-2.299	-6.100
APRICOT	-0.13	0.14	-0.04	0.41	0.18	-0.12	0.74	Fruity & Sweet	9.684	3.982	-1.462
VODKA	-0.16	-0.34	0.35	1.68	-0.04	0.01	0.76	Alcoholic	5.846	-2.383	-9.042
TOFU	-0.17	-0.23	0.26	-1.21	-1.19	-0.86	0.89	Savoury	6.017	-6.255	3.012
KOOL-AID	-0.18	1.17	-0.81	0.14	0.71	NA	NA	Alcoholic	2.706	-3.319	-8.549
TAPA	-0.19	0.75	-0.48	NA	NA	NA	NA	Fruity & Sweet	3.968	-14.281	1.614
PINEAPPLE	-0.20	-0.45	0.45	0.57	0.96	0.58	0.95	Fruity & Sweet	10.083	5.437	-1.940
KEBAB	-0.21	1.12	-0.76	-0.45	0.18	-0.51	0.43	Savoury	5.784	-6.481	6.504
VENISON	-0.21	0.58	-0.34	-0.20	-0.64	-0.33	0.53	Savoury	5.960	-4.584	6.012
HAZELNUT	-0.22	0.75	-0.46	-0.10	-0.74	0.12	0.89	Fruity & Sweet	12.893	2.692	-2.286
RISOTTO	-0.24	1.08	-0.71	-0.24	0.09	0.18	0.81	Savoury	12.678	-4.362	5.282
CHARDONNAY	-0.27	0.58	-0.31	-0.55	0.30	-0.18	0.53	Alcoholic	1.508	-8.510	-2.389
CREOSOTE	-0.27	0.69	-0.39	NA	NA	NA	NA	Stale & Chemical	-9.934	7.165	0.375
MANGO	-0.28	-0.50	0.52	-1.26	0.69	0.14	1.04	Fruity & Sweet	10.077	-0.343	-0.243
SULFIDE	-0.33	-0.86	0.83	NA	NA	-0.08	-1.39	Stale & Chemical	-13.808	-4.316	1.696
MINT	-0.33	-1.07	0.99	1.23	0.67	0.99	1.06	Herbal & Spicy	6.449	9.593	-1.707
AMMONIA	-0.34	-1.50	1.33	-1.33	-1.64	1.75	-1.33	Stale & Chemical	-13.557	2.337	-0.236
CUSTARD	-0.34	0.69	-0.36	-0.65	-0.23	-0.55	0.32	Fruity & Sweet	11.736	-3.047	-1.367
GARNISH	-0.34	0.85	-0.48	-0.38	0.10	-1.39	-0.24	Savoury	8.485	-2.297	3.964
SWEETENER	-0.36	-0.07	0.24	0.21	1.67	-2.13	0.55	Alcoholic	1.407	-5.999	-5.696
VINAIGRETTE	-0.41	1.08	-0.62	NA	NA	0.40	0.71	Savoury	10.482	-1.350	3.162
CHEDDAR	-0.45	0.96	-0.51	NA	NA	-0.45	0.89	Savoury	6.435	-5.055	-0.024
CAYENNE	-0.45	1.08	-0.60	-0.25	-0.15	-0.30	0.63	Herbal & Spicy	6.832	3.768	2.279
COLOGNE	-0.45	-0.43	0.56	-0.52	0.26	1.75	-1.50	Stale & Chemical	-13.482	2.364	-5.350
EXCREMENT	-0.48	0.58	-0.20	0.88	-3.07	0.59	-1.48	Stale & Chemical	-17.233	-7.233	2.134
CURRANT	-0.49	1.12	-0.61	NA	NA	-0.30	0.14	Fruity & Sweet	5.987	5.456	0.668
CHEESECAKE	-0.49	0.82	-0.38	-0.75	1.59	-0.39	1.06	Fruity & Sweet	11.252	-4.828	-2.303
APPETIZER	-0.50	-0.14	0.37	0.72	1.28	0.41	1.03	Fruity & Sweet	4.626	-12.937	4.499
STRAWBERRY	-0.52	-1.31	1.27	0.09	1.25	0.24	1.02	Fruity & Sweet	7.034	2.131	-4.672
ASHTRAY	-0.52	1.04	-0.53	-0.68	-1.82	-0.64	-1.47	Stale & Chemical	-14.646	-6.411	-0.857
BREEZE	-0.52	-1.53	1.45	-1.06	1.54	-2.31	-1.55	Flowery & Fragrant	-10.222	-2.393	3.839
TOBACCO SMOKE	-0.53	-0.17	0.40	1.37	-1.94	0.87	0.07	Stale & Chemical	-18.313	-1.113	0.525
RELISH	-0.54	0.35	0.00	0.63	-0.97	-1.54	-0.32	Savoury	3.462	-8.192	2.185
GINGERBREAD	-0.54	1.17	-0.62	0.26	1.04	0.45	0.46	Fruity & Sweet	-0.725	-2.335	-1.491
HERB	-0.54	-2.10	1.89	-0.98	0.81	0.87	0.80	Herbal & Spicy	6.641	6.526	4.428
YOGURT	-0.56	-0.46	0.64	0.72	1.04	-0.23	0.96	Alcoholic	8.343	-5.767	-4.568
TREAT	-0.56	-0.97	1.04	1.37	0.91	-1.71	0.07	Fruity & Sweet	1.245	-14.912	1.344
GRAPEFRUIT	-0.57	0.27	0.08	-0.41	0.03	-0.06	0.64	Herbal & Spicy	6.727	2.567	-2.233
BLOSSOM	-0.57	-1.07	1.12	1.03	1.08	-0.56	-1.45	Flowery & Fragrant	-3.873	4.671	3.520
BARNYARD	-0.58	1.17	-0.60	-1.93	-0.07	0.12	-1.54	Stale & Chemical	-14.383	-3.010	3.308
HONEY	-0.58	-1.65	1.57	0.53	1.26	-0.02	0.95	Flowery & Fragrant	6.280	4.079	-3.329
SELTZER	-0.59	1.17	-0.60	-1.46	-1.15	-2.05	0.48	Alcoholic	1.819	-5.416	-6.922
CARNATION	-0.60	0.96	-0.43	-0.41	0.01	-0.36	-1.58	Flowery & Fragrant	-5.185	2.360	3.216
POPCORN	-0.61	-0.44	0.66	1.68	1.25	1.22	0.77	Fruity & Sweet	-0.467	-0.698	-2.756
ALMOND	-0.61	-0.13	0.42	-1.48	0.26	-0.63	1.01	Fruity & Sweet	6.456	7.226	-1.514

GELATIN	-0.64	0.00	0.33	0.09	-1.14	-2.16	0.67	Alcoholic	1.863	-4.138	-5.039
CONFECTION	-0.66	1.12	-0.52	NA	NA	-0.39	0.86	Fruity & Sweet	4.752	-4.942	-4.992
DUMPLING	-0.67	0.69	-0.19	-2.00	0.61	-0.38	0.52	Fruity & Sweet	7.404	-8.684	4.655
PEANUT BUTTER	-0.67	-0.11	0.43	-0.68	0.53	-0.48	0.70	Fruity & Sweet	6.488	-1.473	-5.527
MUTTON	-0.68	0.92	-0.36	NA	NA	-0.12	0.46	Savoury	2.289	-5.886	5.176
ROAST	-0.69	-0.50	0.73	0.09	0.01	0.67	0.26	Savoury	2.587	-6.766	5.593
CHLORINE	-0.69	-1.31	1.36	0.60	-1.56	1.22	-0.77	Stale & Chemical	-13.400	-2.249	0.121
ROSE	-0.70	-2.42	2.22	-1.60	1.13	1.06	-1.40	Flowery & Fragrant	-6.030	4.048	2.103
GINGER	-0.70	-0.56	0.79	0.60	0.05	-0.12	0.10	Herbal & Spicy	10.980	5.640	1.944
CIDER	-0.70	0.15	0.25	-0.78	-0.11	0.24	0.84	Alcoholic	3.595	-3.845	-4.521
MOZZARELLA	-0.71	1.08	-0.47	0.75	1.20	-0.96	0.83	Savoury	8.905	-3.160	2.484
SAUSAGE	-0.72	-0.61	0.84	1.10	0.48	0.72	0.83	Savoury	9.094	-3.456	5.077
CHEESE	-0.72	-2.13	2.01	-0.25	0.88	0.25	0.63	Savoury	7.911	-4.812	-0.024
SYRUP	-0.73	-0.65	0.87	-1.18	0.58	-0.21	1.00	Alcoholic	7.273	-1.341	-5.501
PARSLEY	-0.74	0.22	0.21	-1.64	0.43	0.28	0.41	Herbal & Spicy	7.518	4.341	6.267
GRAVY	-0.74	0.20	0.22	-0.18	0.39	0.14	0.67	Savoury	8.650	-7.337	3.779
CHILI	-0.75	0.04	0.35	0.85	-1.05	0.48	0.78	Herbal & Spicy	8.361	-2.875	3.511
CELERY	-0.75	0.31	0.14	-1.58	-0.02	0.08	0.90	Herbal & Spicy	6.766	2.267	6.268
FLOWER	-0.76	-3.29	2.92	-0.43	1.29	1.54	-0.92	Flowery & Fragrant	-5.074	4.213	3.953
REPELLENT	-0.76	-0.27	0.60	0.90	-0.82	-1.44	-1.53	Stale & Chemical	-12.676	-0.956	1.116
SALT	-0.77	-3.01	2.71	0.73	0.26	-1.89	0.95	Herbal & Spicy	0.963	0.371	2.904
ROSEMARY	-0.79	-0.20	0.56	-0.53	0.52	0.75	0.28	Herbal & Spicy	5.472	9.031	4.789
SOUP	-0.80	-1.73	1.74	0.34	1.08	0.36	0.72	Savoury	9.751	-5.267	2.712
BALSAM	-0.81	0.69	-0.11	NA	NA	-1.52	-1.16	Flowery & Fragrant	-4.818	8.570	1.285
ONION	-0.82	-1.49	1.57	1.30	-0.30	1.41	0.92	Herbal & Spicy	6.285	3.156	5.330
TEQUILA	-0.83	0.69	-0.10	2.40	0.44	0.49	1.02	Alcoholic	4.642	-4.070	-7.457
CREAM	-0.84	-2.12	2.06	-0.25	0.68	-0.57	0.77	Fruity & Sweet	6.939	-5.353	-3.906
EUCALYPTUS	-0.85	0.27	0.22	-1.19	0.26	0.37	-0.46	Flowery & Fragrant	-5.106	9.033	2.285
VEAL	-0.85	0.56	0.01	-0.17	-0.76	-0.96	0.37	Savoury	4.943	-8.839	5.500
CHERRY	-0.85	-1.27	1.42	1.25	1.08	-0.48	0.83	Fruity & Sweet	5.703	0.896	-2.237
MILDEW	-0.86	-0.31	0.68	0.21	-2.57	-0.63	-1.58	Stale & Chemical	-17.500	0.453	1.842
PARAFFIN	-0.86	0.88	-0.24	-1.18	-0.52	NA	NA	Stale & Chemical	-14.817	-2.304	3.500
ETHER	-0.86	-0.54	0.86	1.11	-0.91	-0.12	-1.08	Stale & Chemical	-14.657	0.541	-1.436
WINE	-0.87	-3.01	2.76	0.34	0.10	0.78	0.84	Alcoholic	3.899	-5.853	-4.513
LATTE	-0.89	0.96	-0.28	1.08	0.59	0.67	0.88	Fruity & Sweet	1.225	-10.116	-6.054
VERMIN	-0.89	0.88	-0.22	1.66	-2.73	-1.77	-1.33	Stale & Chemical	-19.219	-5.461	5.936
DILL	-0.89	0.85	-0.19	-0.99	0.10	-0.91	0.10	Herbal & Spicy	6.770	7.086	3.877
BRANDY	-0.90	0.31	0.22	-0.17	-0.05	-0.06	0.84	Alcoholic	3.023	0.783	-7.559
SAWDUST	-0.90	0.33	0.21	-1.40	-0.34	-0.90	-1.45	Stale & Chemical	-4.188	7.420	0.955
CAJUN	-0.90	0.92	-0.24	NA	NA	NA	NA	Savoury	5.102	-8.057	5.632
NECTAR	-0.91	-0.03	0.49	-0.05	0.88	-0.84	0.58	Flowery & Fragrant	-4.035	-1.355	-1.290
KETCHUP	-0.91	1.00	-0.30	-0.18	0.24	-0.25	0.92	Savoury	7.574	-6.819	-0.162
MIST	-0.91	-0.96	1.21	-1.26	0.61	-0.75	-0.97	Flowery & Fragrant	-10.269	0.269	1.500
TOOTHPASTE	-0.91	0.19	0.33	-1.52	-0.43	0.24	0.87	Stale & Chemical	-5.036	-1.015	-7.881
POMEGRANATE	-0.93	1.17	-0.42	NA	NA	-0.13	0.92	Fruity & Sweet	3.940	-1.417	2.766
CIGAR	-0.94	-0.81	1.10	0.38	-1.10	0.90	0.10	Stale & Chemical	-6.807	-2.234	-3.738
FRIED CHICKEN	-0.94	1.08	-0.35	NA	NA	NA	NA	Savoury	0.250	-9.555	4.163
SWEAT	-0.95	-1.23	1.44	1.50	-1.11	-0.61	-0.81	Stale & Chemical	-14.678	1.682	-1.900
COSMETIC	-0.97	-1.05	1.31	1.19	-0.53	-2.11	-1.52	Stale & Chemical	-13.666	-8.584	-1.652
BREW	-0.98	0.01	0.49	-0.65	-0.26	-0.38	0.28	Alcoholic	0.980	-9.124	-5.356
COCONUT	-0.98	-0.28	0.72	0.92	0.67	0.56	0.88	Fruity & Sweet	7.614	5.116	-0.062
PISTACHIO	-0.99	1.12	-0.36	0.94	0.29	-1.25	1.13	Fruity & Sweet	11.849	0.318	-2.417
PERSPIRATION	-1.00	0.66	0.00	-0.63	-1.42	-0.85	-1.27	Stale & Chemical	-15.606	-0.729	-1.658
CAVIAR	-1.00	0.85	-0.14	-0.25	-0.17	-0.79	0.19	Fruity & Sweet	4.716	-10.786	0.843
BARBECUE	-1.01	-0.50	0.90	2.11	1.12	1.45	0.97	Savoury	1.986	-7.285	4.478
SWEET	-1.02	-0.93	1.23	0.21	1.67	-1.13	0.72	Fruity & Sweet	3.551	-2.601	-0.808
WHISKEY	-1.02	-0.01	0.53	2.20	-0.15	0.29	0.70	Alcoholic	-2.191	-3.867	-8.405
SOOT	-1.04	-0.14	0.64	0.90	-0.45	-1.48	-1.38	Stale & Chemical	-13.553	-1.117	1.564
SALAD	-1.05	-1.44	1.64	-0.28	0.51	-0.43	1.08	Savoury	11.429	-6.230	3.935
ALE	-1.05	0.17	0.41	0.15	0.18	-0.43	0.53	Alcoholic	3.669	-4.016	-4.368
ESPRESSO	-1.06	0.61	0.08	1.70	0.78	0.46	0.67	Fruity & Sweet	5.083	-5.143	-7.728
SMUT	-1.07	0.78	-0.05	2.20	-1.65	-2.63	-1.54	Flowery & Fragrant	-11.072	-5.137	2.858
STEAK	-1.08	-0.69	1.09	-0.48	0.38	1.01	1.11	Savoury	4.497	-11.788	4.875
PASTRY	-1.08	-0.18	0.69	1.30	0.96	1.43	0.98	Fruity & Sweet	6.202	-7.548	0.097
PICKLE	-1.08	0.12	0.47	-0.68	0.65	0.18	0.96	Savoury	5.332	-2.341	-0.933
GERANIUM	-1.10	0.92	-0.14	NA	NA	-0.61	-1.51	Flowery & Fragrant	-3.776	10.745	5.728
LOTION	-1.11	0.29	0.34	-0.59	-0.18	0.49	-1.37	Stale & Chemical	-7.559	2.666	-5.319
RUM	-1.12	0.12	0.48	1.31	-0.13	0.51	1.07	Alcoholic	6.643	1.537	-8.131

2. Missing data points in the participant data

Supplementary Table 3 shows the percentage of missing data points of each participant variables. These missing data points were imputed in the main analyses.

Supplementary Table 3. Percentage of missing data points of each participant variable.

Variable	% missing data
Age	0%
BMI	12.5%
Sex	0%
olfactory-perceptual ability (TDI)	2.08%
duration impairment	4.17%
degree parosmia	0%
intensity parosmia	0%
valence parosmia	0%
Subjective impairment severity	31.25%
importance of olfaction	4.17%

3. Additional analyses of relationships between behavioral responses and descriptor properties

In order to ensure that our behavioral responses are not driven by word frequency or word concreteness, we also investigated the relationships between qualitatively different and odorless-responses, on the one hand, and log frequency of the descriptors and descriptor concreteness. Concreteness ratings were obtained from Brysbaert et al. (2014). This data set includes ratings of 37,058 words that were rated for their concreteness on a 1–5 point Likert scale. Neither of our behavioral responses correlated significantly with descriptor frequency (*Qualitatively different*: $r = 0.03$, $t(33) = 0.20$, $p = .844$; *Odorless*: $r = -0.27$, $t(33) = -1.64$, $p = .111$) or concreteness ratings (*Qualitatively different*: $r = -0.21$, $t(35) = -1.28$, $p = .209$; *Odorless*: $r = 0.12$, $t(35) = 0.70$, $p = .490$).

4. Principal components analysis

We restrict our analysis to the initial 3 PCs, whose eigenvalues exceed 1, and which individually explain more than 10% of the variance in the data (see Supplementary Figure 4). Supplementary Table 4 shows the proportional contribution of each variable to each of the three components, together with the correlations between variables and the components. In Supplementary Figure 5, the normalized coordinates and contributions of the descriptors of each component are illustrated. The PC coordinates

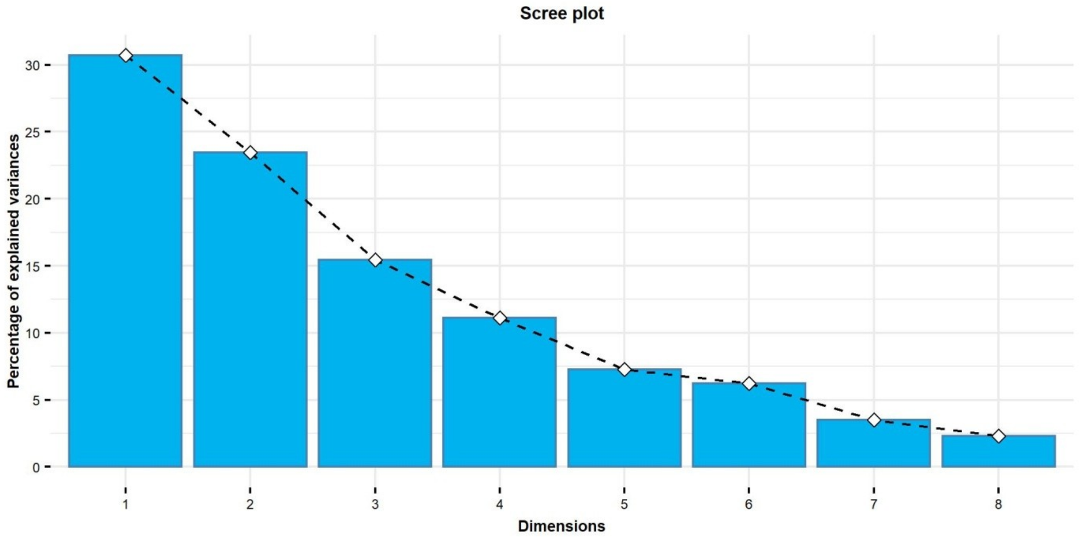

Supplementary Figure 4. Scree plot illustrating percentage of variance explained by the eight principal components of the PCA analysis

Supplementary Table 4. The contribution in % of each variable to each of the three components, and the correlations between variables and the components. Significant effects are marked in grey.

Variable	PC1		PC2		PC3	
	% variance	r	% variance	r	% variance	r
**% Quality difference**	25.51	-0.79	4.73	0.30	8.95	-0.33
**% Odorless**	14.66	0.60	19.90	-0.61	0.72	0.09
**OAI**	2.13	-0.23	1.37	-0.16	44.57	0.74
**OSI**	0.07	-0.04	26.70	-0.71	10.18	-0.35
**Olfactory**	21.77	-0.73	7.09	0.36	3.00	0.19
**Gustatory**	21.04	0.72	16.96	0.56	0.31	-0.06
**Valence**	11.15	0.52	15.84	0.55	9.53	0.34
**Arousal**	3.66	0.30	7.41	0.37	22.74	-0.53

of the descriptors and their relationships to the variables most strongly related to the PCs are illustrated in Supplementary Figure 6 and 7.

PC1 is primarily related to the percentage of *qualitatively different*-responses, the percentage of *odorless*-responses, olfactory and gustatory associations, and to a somewhat lesser extent also to word valence ratings. PC1 is negatively correlated with olfactory associations, but positively correlated with gustatory associations and to a lesser extent with word valence. This suggests that PC1 primarily reflects a gustation-olfaction dimension (Supplementary Figure 6, Panels C & D). However, as edible descriptors primarily refer to pleasant flavors, PC1 also shows a somewhat weaker correlation with word valence (Supplementary Figure 6, Panel E). PC2 is primarily related to the percentage of *qualitatively different*-responses, OSI, gustatory associations and word valence. In particular, PC2 is negatively correlated to OSI (Supplementary Figure 6, Panel F), positively correlated to gustatory associations and word valence (Supplementary Figure 6, Panels C & F), but uncorrelated to olfactory associations (Supplementary Figure 6, Panel D). As such, PC2 seems to be related to descriptor generality, with descriptors being used in a broader range of contexts scoring high (e.g., *coffee*, *wine*, *cheese*). Such descriptors should, in theory, denote a broader and less well-defined range of odor experiences, compared to descriptors that are higher in OSI. As shown by the positive correlation between PC2 and gustatory associations, these descriptors more frequently refer to edibles. Again, as edible descriptors primarily refer to pleasant flavors, PC2 is also positively correlated to word valence (Figure 7 Panel E).

PC3, finally, is related to OAI and arousal ratings (Supplementary Figure 7, Panels A & B), but more or less unrelated to the response variables and all of the other lexical-semantic variables. As such, PC3 seems to reflect the strength with which descriptors are associated to olfactory contexts. There is a tendency for descriptors more frequently used in olfactory contexts to be somewhat lower in arousal, resulting in a negative correlation between PC3 and arousal ratings.

The fact that the percentage of *qualitatively different*-responses is negatively correlated with PC1 indicates that it primarily is odors denoted by descriptors strongly associated with olfaction (e.g., *excrement*, *perfume*, *amonia*) that are percieved qualitatively different (Supplementary Figure 6, Panels A & D). The percentage of *odorless*-responses, on the other hand, is positively correlated with PC1, but negatively correlated with PC2. Taken together, this suggests that it is mostly odors denoted by more specific descriptors (e.g., *pistachio*, *mothball, gingerbread*) that are harder to perceive overall; they are less strongly associated with olfaction, and therefore to some extent more strongly associated with gustation. (Supplementary Figure 6, Panels A & F).

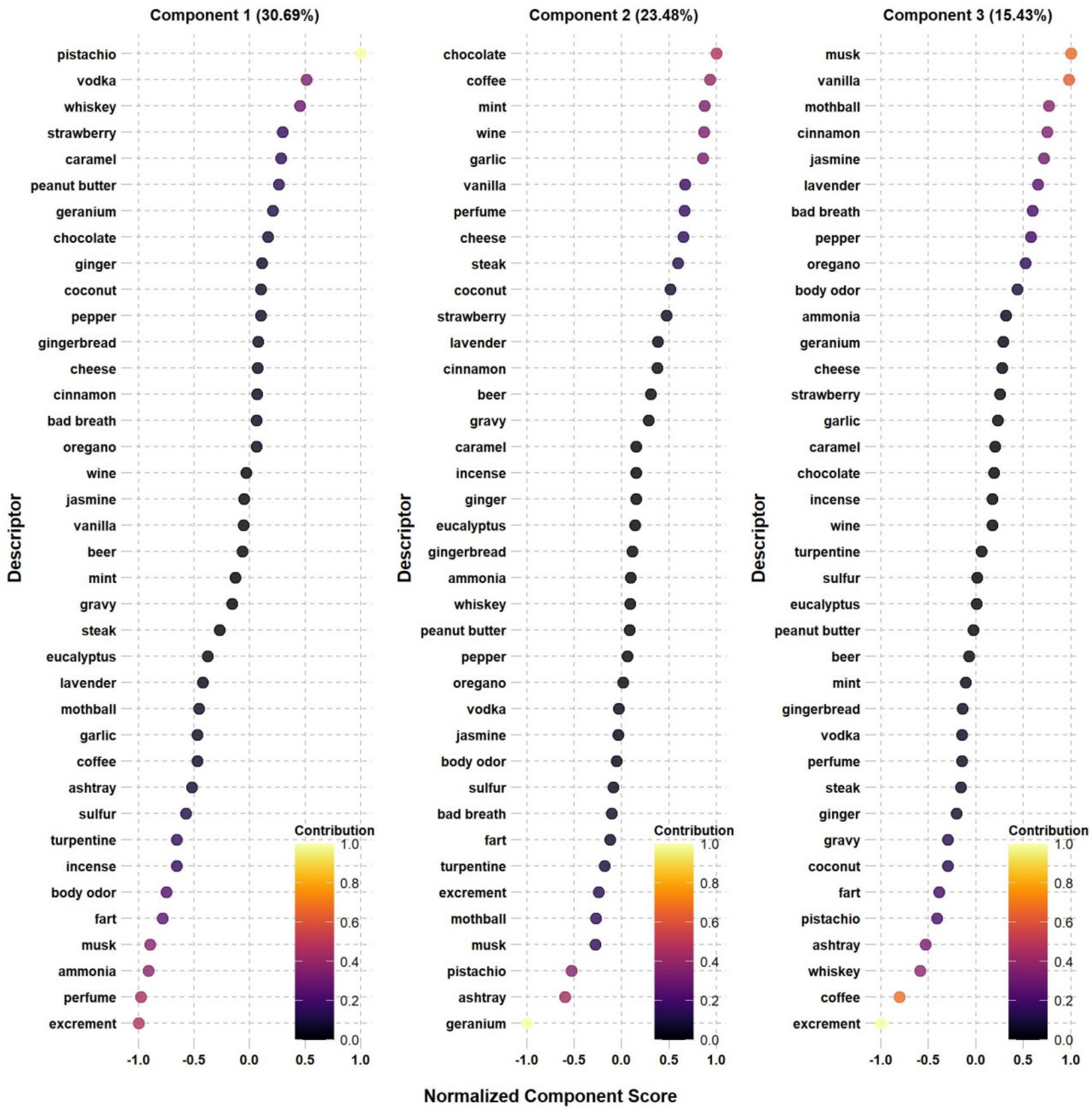

Supplementary Figure 5. Normalized component coordinates and contributions of the descriptors of each component.

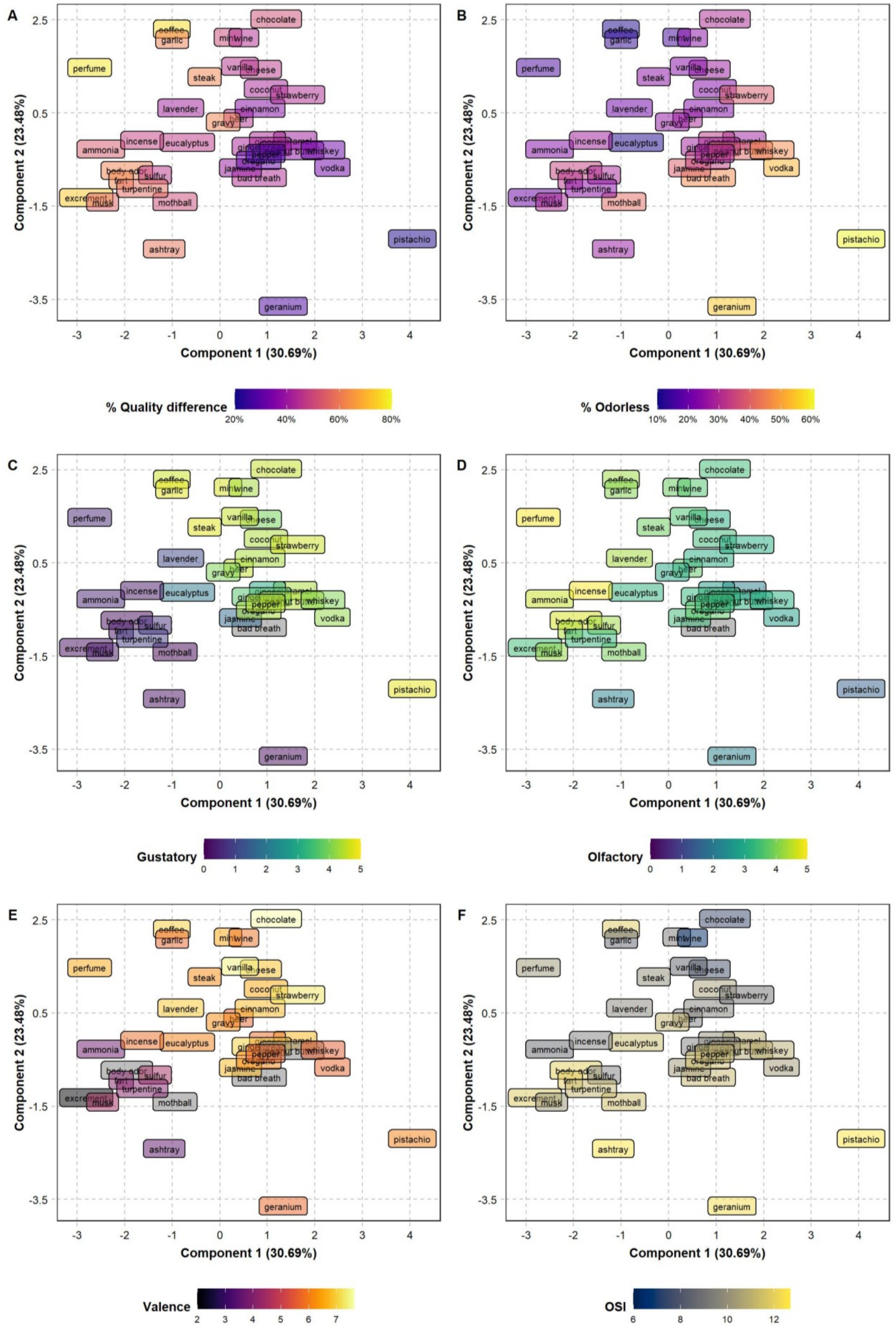

Supplementary Figure 6. Descriptor positions on PC 1-2 and their relationships to the variables of interest. A) Quality difference. B) Odorless. C) Gustatory association. D) Olfactory association. E) Valence. F) OSI.

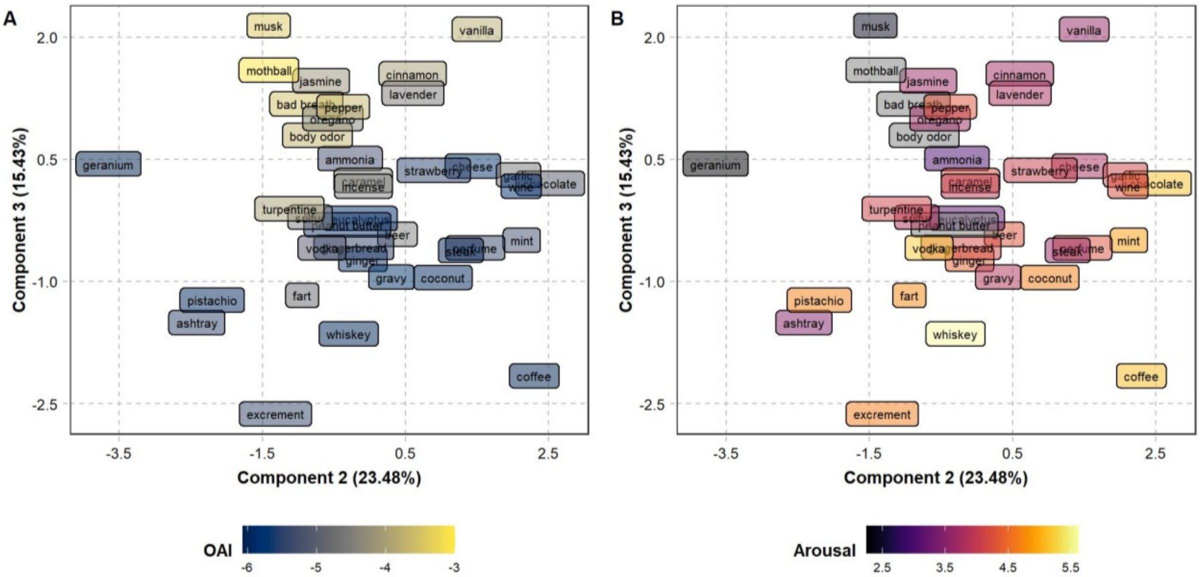

Supplementary Figure 7. Descriptor positions on PC 2-3 and their relationships to the variables of interest. A) OAI. B) Arousal.

References

Brysbaert, M., Warriner, A. B., & Kuperman, V. (2014). Concreteness ratings for 40 thousand generally known English word lemmas. Behavior Research Methods, 46(3), 904–911. https://doi.org/10.3758/s13428-013-0403-5

Han, L., Kashyap, A. L., Finin, T., Mayfield, J., & Weese, J. (2013). *UMBC_EBIQUITY-CORE: Semantic Textual Similarity Systems*. 44–52.

Holm, S. (1979). A Simple Sequential Rejective Multiple Test Procedure. *Scandinavian Journal of Statistics*, *6*, 65–70.

Hörberg, T., Larsson, M., & Olofsson, J. K. (2020). *Mapping the semantic organization of the English odor vocabulary using natural language data* [Preprint]. https://psyarxiv.com/hm8av/

Kaufman, L., & Rousseeuw, P. J. (1990). *Finding Groups in Data*. John Wiley & Sons, Inc. https://doi.org/10.1002/9780470316801

Mikolov, T., Chen, K., Corrado, G., & Dean, J. (2013). Efficient Estimation of Word Representations in Vector Space. *ArXiv:1301.3781 [Cs]*. http://arxiv.org/abs/1301.3781

Maechler, M., Rousseeuw, P., Struyf, A., Hubert, M., & Hornik, K. (2019). *cluster: Cluster Analysis Basics and Extensions* (R package version 2.1.0) [R].

R Core Development Team. (2018). *R: A Language and Environment for Statistical Computing*. R Foundation for Statistical Computing. http://www.R-project.org/
